# Mathematical Model of Evolution of Brain Parcellation

**DOI:** 10.3389/fncir.2016.00043

**Published:** 2016-06-16

**Authors:** Daniel D. Ferrante, Yi Wei, Alexei A. Koulakov

**Affiliations:** Cold Spring Harbor LaboratoryCold Spring Harbor, NY, USA

**Keywords:** evolution, brain regions, brain regionalization, whole brain connectivity, connectome, wiring diagram, parcellation map

## Abstract

We study the distribution of brain and cortical area sizes [parcellation units (PUs)] obtained for three species: mouse, macaque, and human. We find that the distribution of PU sizes is close to lognormal. We propose the mathematical model of evolution of brain parcellation based on iterative fragmentation and specialization. In this model, each existing PU has a probability to be split that depends on PU size only. This model suggests that the same evolutionary process may have led to brain parcellation in these three species. Within our model, region-to-region (macro) connectivity is given by the outer product form. We show that most experimental data on non-zero macaque cortex macroscopic-level connections can be explained by the outer product power-law form suggested by our model (62% for area V1). We propose a multiplicative Hebbian learning rule for the macroconnectome that could yield the correct scaling of connection strengths between areas. We thus propose an evolutionary model that may have contributed to both brain parcellation and mesoscopic level connectivity in mammals.

## Introduction

The brain has many distinct regions defined anatomically and functionally. The evolutionary origin of the diversity of brain regions is not well understood (Kaas, 2009). According to one theory, new brain regions emerge from existing ones through the process of fragmentation and specialization (Striedter, 2004; Kaas, 2009). Due to the abundance of brain regions, fragmentation is expected to be an iterative process persisting through brain evolution. One can infer properties of this process from the distribution of resulting fragments, i.e., brain region sizes. Here we examine the distributions of brain region sizes, called here parcellation units (PU), for three species: mouse, macaque, and human. We infer parameters of the brain fragmentation process that can lead to these distributions. We argue that brain fragmentation followed a similar evolutionary mechanism in the three species analyzed.

Interestingly, the problem of brain parcellation is mathematically related to the fragmentation of shells of explosive projectiles and warheads as well as to rock grinding and crushing, described previously in Epstein (1947), Grady and Kipp (1985), and Redner (1989). In explosive shell and rock fragmentation, one original piece gives rise to a distribution of fragments that independently undergo further crushing. The class of processes with independent fragmentation was first analyzed by Kolmogorov (1941). Kolmogorov showed that under appropriate conditions, sequential breakage yields a lognormal distribution of particle sizes (mathematician was intrigued by the lognormal distribution of gold particles Kolmogorov, 1941). The continuous crushing of rocks and explosive shells, just like the repeated subdividing of brain regions, results in an evolving distribution of cluster sizes, which can yield information about the underlying process. In the present study, we both evaluate the statistical distribution of brain region sizes and propose an evolutionary model that is somewhat distinct from Kolmogorov's theory. We also study the implications of brain evolution by fragmentation for region-to-region connectivity.

## Methods

We used MATLAB (Mathworks, Inc.) to perform computer simulations and fits as described in the text. To implement our parcellation model, we computed probabilities of the regions to be split, according to Equation (1), normalized it for the entire existing ensemble, and, using this set of probabilities, selected one PU randomly on each iteration of the algorithm. This PU was then split into two equal parts, producing two new PUs. After two new PUs were generated, their volumes were multiplied by a random variable (normal distribution, mean equals to 1, 10% standard deviation) in such a way that their total volume is preserved. This was done to make volumes of PUs deviate from the exact powers of 1/2. We verified that the inclusion of multiplicative noise did not affect noticeably our results. These steps were repeated until a desired number of PUs was generated.

## Results

### The distribution of PU sizes is close to lognormal

**Figure 1:**
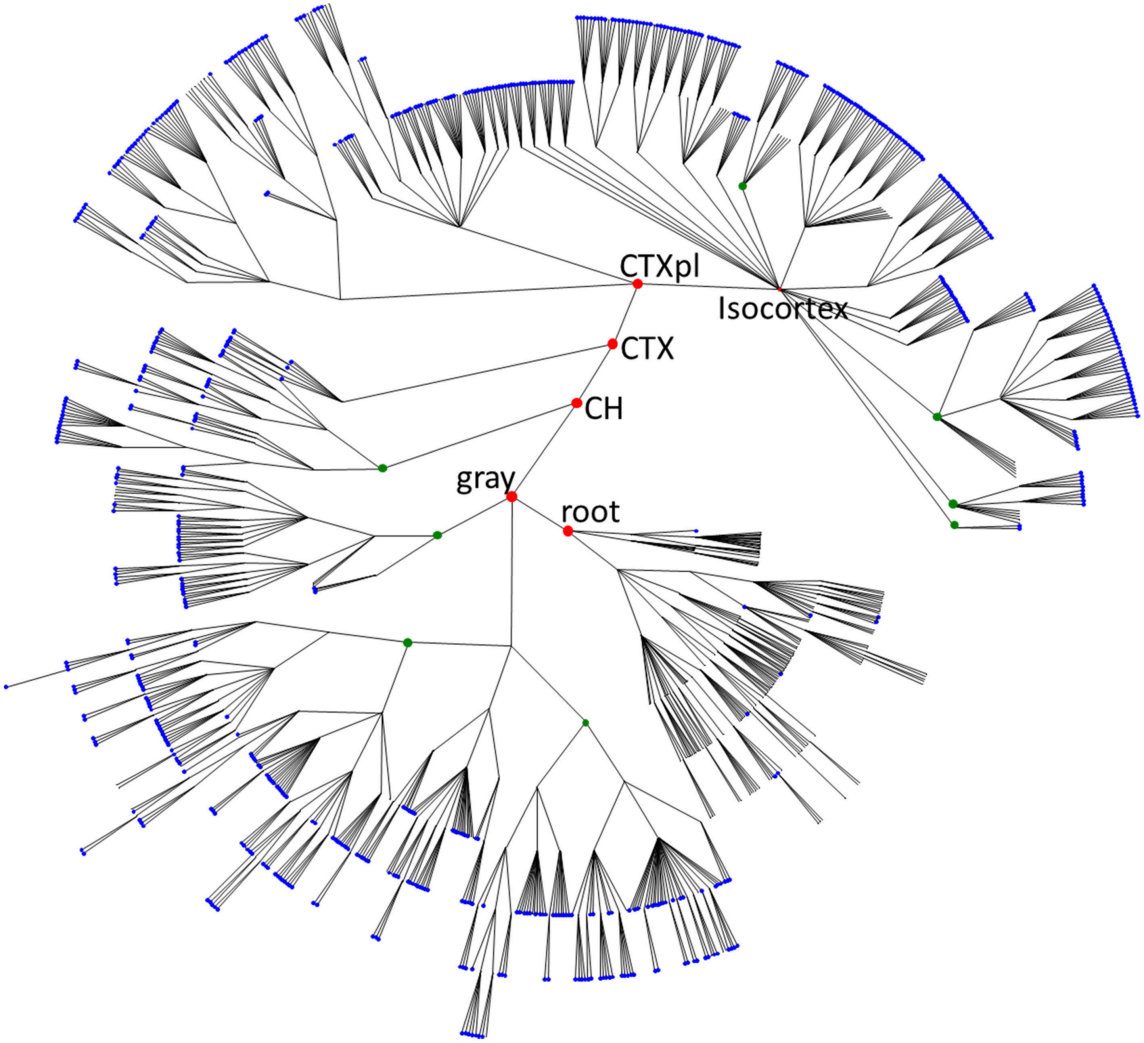
**The overview of mouse brain parcellation tree**. The tree is based on Allen Brain Institute reference atlas anatomical structure hierarchy (Dong, 2008; Osten and Kim, 2013). Each brain region (PU) is represented by a node. Root node represents the entire brain of the animal. Empty nodes represent branch points at which division of a PU into smaller PUs occurs. Blue nodes designate leaves of the tree with non-zero volumes that are included into the histogram in Figure 2A. Green nodes show some binary branch points (duplications). In our model, we assume that non-binary branch points corresponding to fragmentations into three or more PUs (empty branch points) represent a series of duplications that have occurred at different times. Red nodes represent major brain structures annotated in the Figure (root, the root of the tree; gray, basic cell groups and regions; CH, cerebrum; CTX, cerebral cortex; CTXpl, cortical plate).

**Figure 2:**
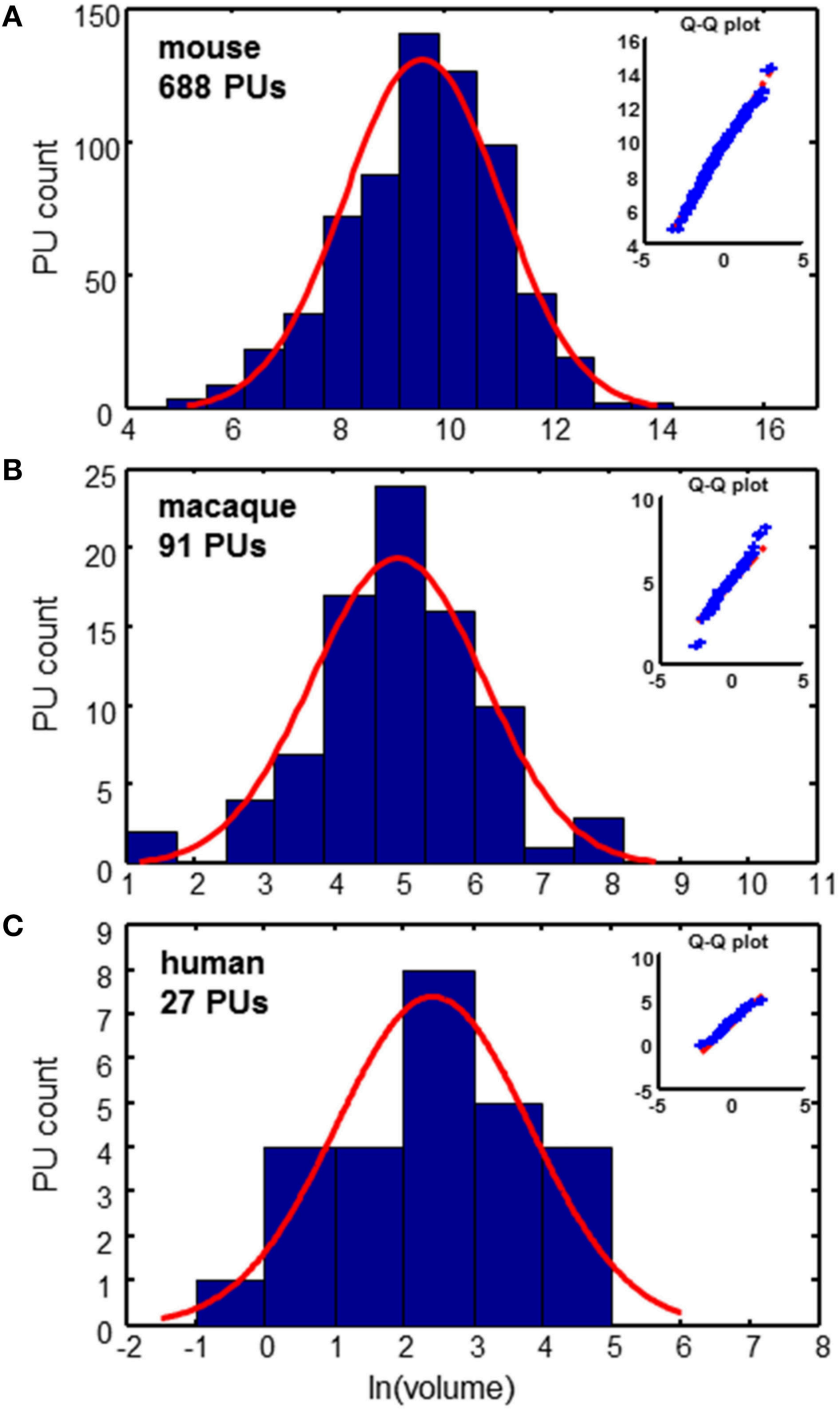
**Distributions of PU volumes are close to lognormal**. Red curves show Gaussian fits. Q-Q plots indicate the high quality of fits. **(A)** Mouse brain: σ = 1.47 **(B)** Macaque cortex: σ = 1.24 **(C)** Human cortex: σ = 1.40. Units of volume: Number of voxels (mouse), mm^3^ (macaque), and cm^3^ (human).

PU volume data is available for the three species: mouse (Dong, 2008; Osten and Kim, 2013), macaque (Markov et al., 2011, 2014; Bakker and Kötter, 2016), and human (Mai et al., 2007). For the mouse data, it is possible to reconstruct the tree formed by the segmentation of the brain into PUs, as shown in Figure 1. To analyze the distribution of PU sizes, we fit the distributions of the logarithm of brain region volumes for all three species with Gaussian distributions. Overall, the distributions appear to be close to normal, suggesting that the distribution of PU sizes is close to lognormal (Figure 2). The standard deviations of the logarithm of PU sizes are similar for all three species (σ = 1.47, 1.24 and 1.40 of natural logarithm units for mice, macaques and humans respectively). Statistical tests [Kolmogorov-Smirnov (KS) (Massey, 1951)] and quantile-quantile (QQ) plots (Figure 2) show that the distributions of the logarithms of PU size are close to normal (*p*_*KS*_ = 0.07, 0.48, 0.97 for the three species). Larger *p*-values indicate that the observed distributions of brain region sizes are close to lognormal distributions.

### The evolutionary parcellation model

**Figure 3:**
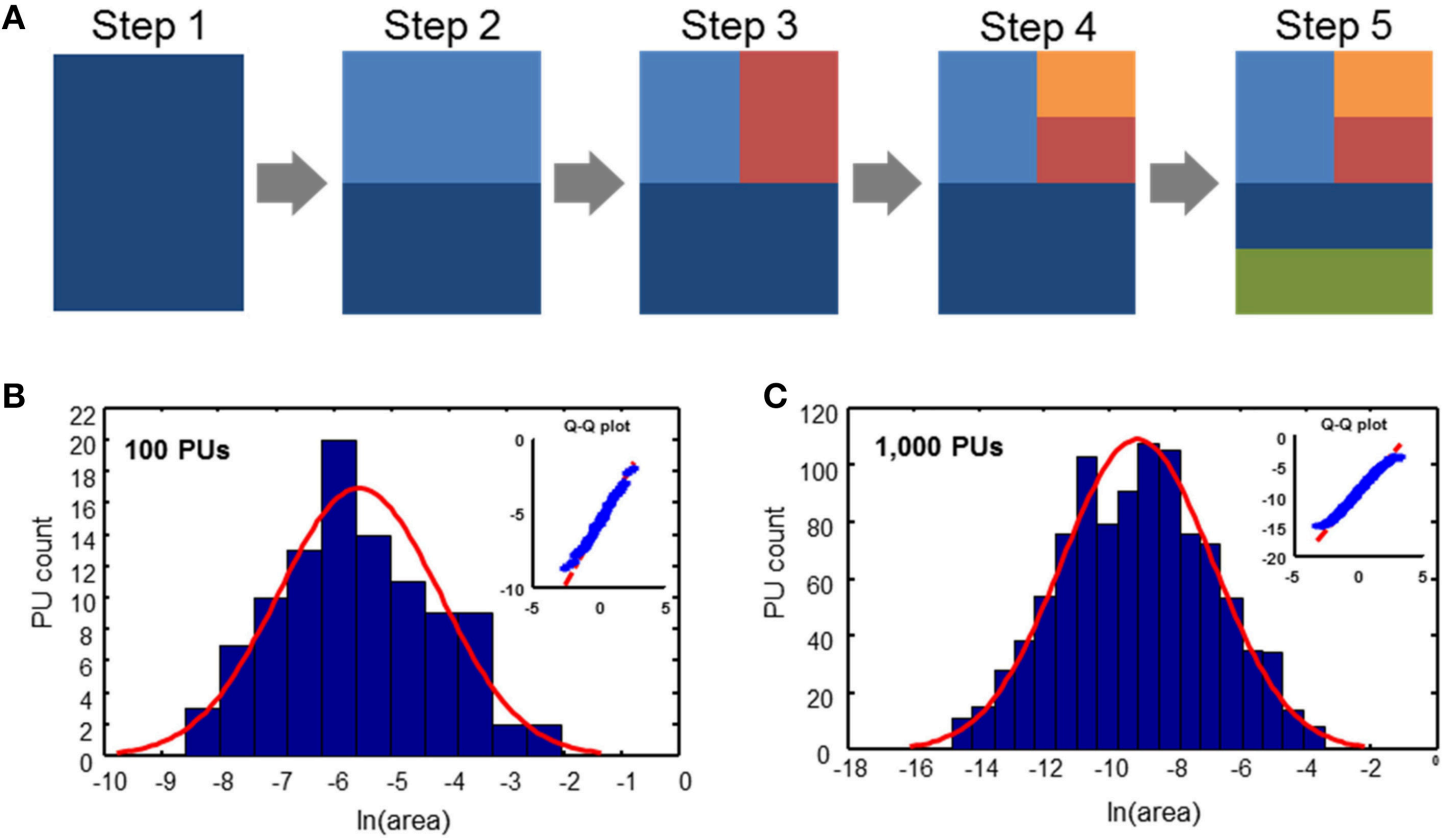
**Evolutionary parcellation model yields distributions close to lognormal. (A)** A parent region is chosen randomly and divided into two new regions. This process is repeated until a target number of PUs is reached. **(B,C)** Distributions of PU sizes generated by the model. **(B)** 100 PUs: σ = 1.40; *p*_*KS*_ = 0.93; **(C)** 1000 PUs: σ = 2.31; *p*_*KS*_ = 0.33. In these and subsequent simulations, immediately after a division, two divided regions are varied in size by 10% randomly, while maintaining the sum of their volumes constant. This was done to produce region sizes different from fractions of 2.

We propose a simple evolutionary parcellation model that can explain these observations. Our process starts with a *tabula rasa* brain containing only a single region (Figure 3A, step 1). This region is then divided into two PUs of equal size (step 2). In the next step, we choose one of these two regions with equal probability and divide it again into two equal parts (step 3). This sequence of steps, including picking a random region independently of its size and dividing it, is repeated until the target number of PUs is achieved (Figure 3). Iterating this model results in a distribution of brain region sizes which is close to log-normal (Figure 3).

### The biased parcellation model

The model presented above generates new PUs by randomly selecting a region to be fragmented into two new regions of similar size. In this model, which we call the model of uniform parcellation, all PUs had the same probability to be split independently on their volume. We showed that this model leads to a distribution of PU volumes that is close to lognormal (Figure 3), as observed in the experimental data (Figure 2). Is it possible that the regions are selected to be fragmented according to their size? To answer this question, we formulated the model, in which the fragmentation probability is a function of the PU volume. We assumed that the probability of fragmenting a region number *i* at a given time step is described by the power law distribution

(1) $$\begin{array}{l} p_{i}\;=\;v_{i}^{\mu }∕Z. \end{array}$$

Here $v_{i}^{\mu }$ is the volume of PU number *i*, *Z* is the normalization coefficient, while μ is a parameter (exponent) of the model. We assumed the power law function in equation (1) because it allows defining probability for a wide range of PU volumes spanning several orders of magnitude. For μ = 0, the probability of dividing a PU is independent of its size, which describes the model of uniform parcellation we considered before (Figure 3). For positive values of μ, larger regions are more likely to be split, leading to a brain containing PUs of more similar size. For negative μ, smaller PUs are expected to be split more often, leading to a wide distribution of PU sizes. The more general mechanism described by equation (1) will be called here the model of biased parcellation.

**Figure 4:**
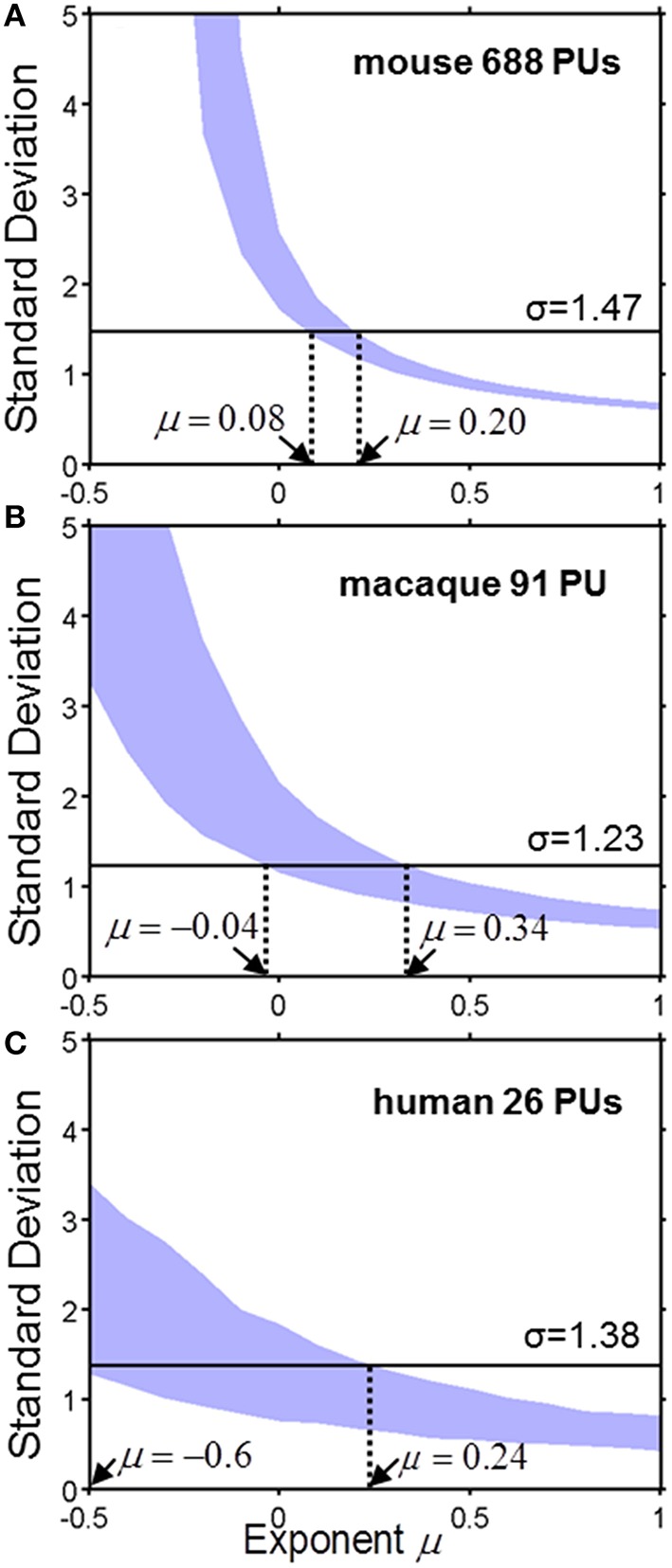
**The standard deviation σ of the logarithm of PU size as a function of exponent μ for the mouse (A), macaque (B) and human (C)**. The shaded region represents a 90% confidence interval for σ, which was obtained from a sample of 1000 simulations for each species with the specified number of PUs. The horizontal line describes the observed value of μ. Ranges consistent with the model are indicated by the dotted lines. **(A)** Mouse brain. 0.08 ≤ μ ≤ 0.20. **(B)** Macaque cortex: −0.04 ≤ μ ≤ 0.34. **(C)** Human cortex: −0.6 ≤ μ ≤ 0.24.

To analyze quantitatively the parcellation process for various values of μ we compute the standard deviation of the logarithm of PU sizes as a function of μ (Figure 4). The standard deviation defines the width of a PU size distribution. The width of a distribution is dependent on the number of PUs. Indeed, since, on every time step, our model increases the number of PUs by one, the number of PUs determines the number of time steps needed to evolve a given set of brain structures. Longer evolution leads to a larger diversity in PU sizes, since PU distribution is determined by a diffusion-like process. A simple calculation shows that, for μ = 0, for example, the variance of logarithms of PU sizes depends on time as σ^2^(ln *v*) ~ ln *t*, where evolutionary time is measured by the number of PUs, *t* = *N*_*PU*_. For any given μ, therefore, the diversity of PU sizes can depend on the number of PUs. The three panels in Figure 4 present the dependence of σ(ln *v*) on the exponent μ for the number of PUs *N*_*PU*_ matching the three species for which the data is available (Figure 2). By obtaining the range of μ consistent with the σ(ln *v*) observed in each of the species, one can infer the exponent guiding brain parcellation in these animals. The shaded region in Figure represents a 90% confidence interval for the values of μ, obtained from a sample of 1000 simulations for each data set. The 90% confidence intervals for μ overlap in the three species within the interval 0.08 ≤ μ ≤ 0.20. This suggests that the evolution of brain parcellation may have followed a similar rule in the three organisms, with the selection of next region for fractioning slightly biased toward larger PUs (μ > 0). The model of uniform parcellation described above (Figure 3) is therefore close to the model of parcellation with a bias.

### Outer product form of region-to-region connectivity

The evolutionary parcellation model described here has implications for the connectivity strengths between different brain regions. Such connectivity is defined through the total number of wires running between a pair of regions and is conventionally called the macroconnectome, to distinguish it from the connectivity with a single-neuron precision, i.e., the microconnectome. In the simplest parcellation model of the macroconnectome, brain evolution starts from the tabula rasa state in which every two neurons have a finite probability *f* to be connected. The total number of connections is therefore equal to *C* = *fN*^2^∕2, where *N* is the total number of neurons and *N*^2^ ∕ 2 is the total number of pairs. After the parcellation process has run its course, the number of connections between any two areas with volumes *v*_*i*_ and *v*_*j*_ is expected to be $C_{ij}\;=\;v_{i}f\rho^{2}\;v_{j}∕2$. Here ρ is the number density of neurons, assumed to be the same for all areas (Carlo and Stevens, 2013). Within the parcellation model, the macroconnectome defined by matrix *C*_*ij*_ is therefore expected to have the outer product form, i.e., *C*_*ij*_ ∝ *v*_*i*_*v*_*j*_. This simple theory generates an experimentally testable prediction. In particular, it predicts that the strength of connections for one of the brain regions, number *i*, for example, with an array of other regions, numbered by index *j*, should be proportional to the size of these regions *v*_*j*_, which is a consequence of the outer product form of the connection matrix *C*_*ij*_ ∝ *v*_*i*_*v*_*j*_.

**Figure 5:**
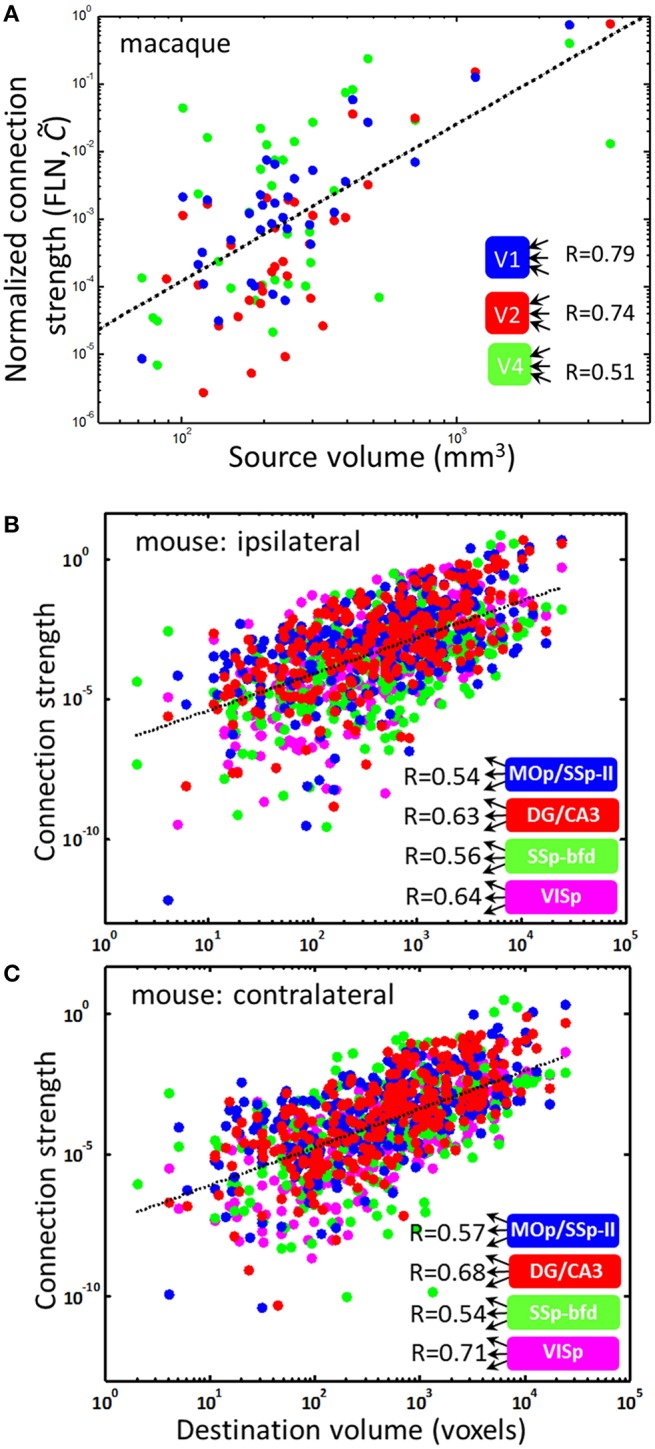
**PU volumes contain information about connection strengths**. The dependences of connection strength on PU size. **(A)** Non-vanishing (${\tilde{C}}_{ij}\neq 0$) incoming connection strengths for three target visual areas in the macaque brain and various other areas as sources. The linear fit (dotted line) has a slope of η = 2.33. Individual slopes for the three areas V1, V2, and V4 are η = 2.67, 2.85, and 1.79. The scaling law ${\tilde{C}}_{ij}\propto v_{j}^{\eta }$ can explain the fractions *R*^2^ = 0.62, 0.54, and 0.25 data variance for these areas. **(B,C)** Non-vanishing outgoing connections in the mouse brain for four source regions are described by a similar scaling relationship $C_{ij}\propto v_{i}^{\kappa }$, where the exponent κ = 1.30 and 1.35 for ipsi- and contralateral connections is obtained from the linear fits (dotted lines).

To test the outer product form of the macroconnectome, we analyzed the dependence of the number of connections between three macaque visual areas, V1, V2, and V4, and an array of 91 cortical areas. The data was available from a recent study of the distribution of the number of connections (Markov et al., 2011). This data contained the number of neurons projecting to a given target area obtained using retrograde tracing of connections. To eliminate uncertainties associated with the dimensions of tracer injection, the number of connections was normalized to one. Thus the data is represented by a normalized connection matrix ${\tilde{C}}_{ij}\;=\;C_{ij}∕\underset{k}{\sum }C_{ik}$, called the fraction of labeled neurons (FLN). It is easy to see that $\underset{k}{\sum }{\tilde{C}}_{ik}\;=\;1$. If the original connection matrix *C*_*ij*_ is given by the outer product form *C*_*ij*_ ∝ *v*_*i*_*v*_*j*_, as our model suggests, the FLN matrix is proportional to the volume of the source, i.e., ${\tilde{C}}_{ij}\propto v_{j}$. We therefore plotted the logarithm of connection strength ${\tilde{C}}_{ij}$ as a function of the logarithm of source volume *v*_*j*_ expecting to observe a positive correlation. Indeed, we find that for all three target areas, V1, V2, and V4, the logarithm of normalized non-zero connection strength is correlated with the logarithm of source area volume (Figure 5A). For the area V1, for example, the correlation is close to *R* = 0.78, suggesting that *R*^2^ = 62% of the connectivity data is explained by the source area size. For other areas the correlation is weaker, however, and data for these areas appears to be more variable due to fewer injections (the number of injections yielding connectivity was 5, 4, and 3 for V1, V2, and V4 in Markov et al. (2011), producing *R*^2^ = 0.62, 0.54, and 0.25 respectively).

### The Hebbian model for outer product form of connectivity

Although area-to-area connectivity appears to be close to the outer product form (*R*^2^ = 0.62), the slope of dependences in Figure 5A is not consistent with the simple parcellation model presented above. Indeed, although linear fits to dependences in Figure 5A account for large amounts of data (R^2^), these linear fits result in a power law relationship between the area-to-area connection strength and source volume, i.e., ${\tilde{C}}_{ij}\propto v_{j}^{\eta }$, with η = 2.67, 2.85, and 1.79, respectively, for V1, V2, and V4. The exponent obtained from fitting combined data for V1, V2, and V4 is η = 2.33 (*R* = 0.63). In contrast, the simple parcellation model for connectivity presented above yields η = 1 (${\tilde{C}}_{ij}\propto v_{j}$). Thus, although the simple model predicts the outer product form of area-to-area connectivity and the power-law dependence of connection strength on PU volume (${\tilde{C}}_{ij}\propto v_{j}$), the exponent in the law is not captured exactly; the observed value of the exponent is larger. This is expected, however, since the macroconnectome can be modified to be better suited for the particular computations relevant for an organism's survival after the areas are split. Nonetheless, a substantial amount of data on non-zero macroconnectivity (as much as 62% for area V1) can be explained by the source area size through the power law described above.

To gain insight into the mechanism of emergence of the power law relationship between the number of area-to-area connections and the source area size, we propose a model that is based on a Hebbian learning rule. It was recently proposed that the microconnectome (connections within a cortical column) is determined by the scale-free multiplicative Hebbian learning rules of the form (Koulakov et al., 2009)

(2) $$\begin{array}{l} dC_{ij}∕dt\;=\;\varepsilon_{1}f_{i}^{\alpha }\;C_{ij}^{\beta }\;f_{j}^{\gamma }\;-\;\varepsilon_{2}\;C_{ij}. \end{array}$$

Here, *f*_*i*_ is the activity level in area number *i*, the first term describes the correlations in neural activity, the second term in the r.h.s. is the connection decay, ε_1_ and ε_2_ are constant parameters, while α, β, and γ are exponents. By assuming that connection strengths have already reached equilibrium for a given activity configuration (*dC*_*ij*_ ∕ *dt* = 0) and that the overall activity levels are proportional to the area size (*f*_*i*_ ∝ *v*_*i*_), we obtain $C_{ij}\propto v_{i}^{\alpha ∕\left(1-\beta \right)}v_{j}^{\gamma ∕\left(1-\beta \right)}$, i.e., the outer product power-law form of connectivity with η = γ∕(1 − β) and κ ≡ α∕(1 − β). To match our observations for area V1, we have to assume that η = γ∕(1 − β) ≈ 2.67. Thus, the same Hebbian learning rule postulated in Koulakov et al. (2009) can be used to derive both micro- (within area) and macroconnectivity (area-to-area).

### Outer product form of outgoing connections

The outer product form can also be tested for the outgoing connections, i.e., $C_{ij}\sim v_{i}^{\kappa }$. Recent studies make available outgoing connectivity strengths for 295 target mouse brain regions (Oh et al., 2014). This data suggests that the outer product form of connectivity describes *R*^2^ = 30–50% of connection data with an exponent κ ≈ 1.3 (Figures 5B,C). Data in Figures 5B,C are presented for four regions with ≥ 3 injections. Although Figure 5 includes only non-zero connections, it is notable that the Hebbian learning rule (2) includes zero connections (*C*_*ij*_ = 0) as a solution, thus capturing both vanishing (*C*_*ij*_ = 0) and non-vanishing (*C*_*ij*_ ≠ 0) connections.

## Discussion

Here, we have studied an evolutionary model for the emergence of a distribution of brain regions (PUs) according to their size. We aimed at describing the ensemble of brain regions statistically, without addressing the functional significance of individual PUs. We assumed that brain regions emerge through a sequential process of fragmentation and specialization. Kolmogorov's fragmentation model (Kolmogorov, 1941) assumes that the splitting process for each fragment occurs independently of other pieces. This model is appropriate to describe explosive shell fragmentation or rock grinding (Epstein, 1947; Grady and Kipp, 1985; Redner, 1989). For such a process, under conditions of stationary parameters, the total number of pieces grows exponentially. Since there is no evidence for the explosion in the number of brain regions that occurred recently, we were motivated to find a different model that would describe a more gradual proliferation of PUs, one at a time.

We find that, similarly to Kolmogorov's model, the distribution of PU sizes in our model is close to lognormal. The variance of the logarithm in our model depends on the scaling of the splitting probability with volume (Equation 1). We find that in all three species, mouse, macaque, and human, brain/cortex parcellation is consistent with a scaling exponent within the range 0.08 ≤ μ ≤ 0.20, suggesting that common evolutionary mechanisms may have shaped the brains of these animals. It is conceivable that each PU undergoes multiplicative variations in size between fragmentations, which should slightly increase the values of μ. We find therefore that the probability of PU fragmentation is dependent on PU size (Equation 1), by contrast with the Kolmogorov theory.

Factors other than studied here may have contributed to the diversity of brain region volumes. Indeed, PUs may continue to expand or contract as a result of continuing trend inherited from their parent PUs and determined by their functional properties. PUs may undergo changes in relative volumes as a result of their divergence in functional properties occurring after fragmentation. The fragmentation may yield two PUs of different sizes (our model includes this effect to a small degree to make PU volumes deviate from exact powers of 2, see Methods). Ensemble wide data on relative significance of these and other effects is not available. One can speculate, however, about potential effects of these factors on the diversity of PU volumes. It seems obvious that including additional sources of variability in the model, such as uneven duplications or ongoing changes in PU volumes, should increase the width of the final distribution of PU sizes. Thus, the curves in Figure 4 should be located at higher values of standard deviation. This, in turn, will increase the estimates of the parameter μ. Thus, it seems that including additional sources of variability into our model, should make it trend further away from the model of uniform parcellation. These effects should introduce a higher bias in the selection of PU to be fragmented toward larger regions. Quantitative accounting for these effects would require detailed comparison of parcellation trees in related species.

We analyzed the dependence of area-to-area (macro) connectivity in the macaque cortex and its relation to PU sizes that was suggested by our parcellation model. We found that the incoming connectivity is well described by the outer product power-law form with similar scaling relationships for three visual areas: V1, V2, and V4. Remarkably, we find that for area V1, 62% of information about its non-zero incoming connection strengths to other areas is contained in the simple scaling relation ${\tilde{C}}_{ij}\propto v_{j}^{\eta }$, with the scaling exponent η > 1. Macro connection strength is therefore mostly determined by the sizes of connected regions. The lognormal distribution of connection strengths (Markov et al., 2011) may therefore result from the lognormal distribution of PU sizes, reported here. The remaining 38% of V1 connectivity may be attributed to non-outer product features, such as connection locality (Klyachko and Stevens, 2003; Markov et al., 2011), as well as important functional requirements of the macroconnectivity that do not have an outer product structure. The outer product form of connectivity presented here could serve as a baseline model for brain-wide connectivity with deviations from the outer product form specifying the importance of individual connections. One cannot rule out the contribution to the unexplained fraction of connectivity from the residual noise in the data. We find that the quality of power law fits (${\tilde{C}}_{ij}\propto v_{j}^{\eta }$) is dependent on the number of injections into targets, suggesting that more reliable data could improve fit quality. We proposed a Hebbian learning paradigm that could explain the outer product form of macroconnectivity. We thus proposed a universal evolutionary law that could both guide the formation of brain regions and contribute substantially to their connectivity. This law generalizes across several mammalian species.

## Author contributions

All authors listed, have made substantial, direct and intellectual contribution to the work, and approved it for publication.

### Conflict of interest statement

The authors declare that the research was conducted in the absence of any commercial or financial relationships that could be construed as a potential conflict of interest.
